# Supplementary material for: Behavioral responses of pyrethroid resistant and susceptible Anopheles gambiae mosquitoes to insecticide treated bed net
Source: PLoS One. 2022 Apr 7;17(4):e0266420. doi: 10.1371/journal.pone.0266420 (PMC8989192; doi:10.1371/journal.pone.0266420)
Supplement: S1 Table — (DOCX) [file pone.0266420.s001.docx]

**S1 Table. Results on the fate of all released mosquitoes of each strain when an insecticide treated and untreated panel was present.**

| Status of Bednet trap | Mosquito population | No. released | No. trapped in Mbita trap | No. resting indoor No. Exiting | No. Exiting | No. resting outdoor | No. House entry | No. Recaptured | No. Not recaptured |
| --- | --- | --- | --- | --- | --- | --- | --- | --- | --- |
| Treated | Resistant | 3000 | 954 | 501 | 79 | 104 | 1534 | 1638 | 1362 |
|  | Susceptible | 3000 | 426 | 343 | 424 | 544 | 1193 | 1737 | 1263 |
| Untreated | Resistant | 3000 | 882 | 640 | 42 | 64 | 1564 | 1628 | 1372 |
|  | Susceptible | 3000 | 935 | 600 | 43 | 82 | 1578 | 1660 | 1340 |
| Treated | Wild population( F1) | 1000 | 169 | 188 | 80 | 67 | 437 | 504 | 496 |
| Untreated | Wild population( F1) | 1000 | 211 | 237 | 18 | 43 | 466 | 509 | 491 |
