# Supplementary material for: Behavioral responses of pyrethroid resistant and susceptible Anopheles gambiae mosquitoes to insecticide treated bed net
Source: PLoS One. 2022 Apr 7;17(4):e0266420. doi: 10.1371/journal.pone.0266420 (PMC8989192; doi:10.1371/journal.pone.0266420)
Supplement: S2 Table — (DOCX) [file pone.0266420.s002.docx]

**S2 Table. Results from the mixed linear model fit by maximum likelihood examining the impact of bednet status and number of mosquitoes released, while considering night as a random effect, on the number of mosquitoes trapped during the night.**

| **Fixed Effects** |  | **Estimate** | **Estimates 95% Cl** | **t value** | **Pr(>\|t\|)** |
| --- | --- | --- | --- | --- | --- |
| (Intercept) |  | 51.86 | 38.9 – 64.7 | 8.09 | < 0.001 |
| Number _mosquito released |  | 0.1239 | 0.0882 – 0.1596 | 6.98 | < 0.001 |
| Bednet Status (Treated vs.Untreated) |  | -27.62 | -34.6 – -20.7 | -8.02 | < 0.001 |
