# Supplementary material for: Behavioral responses of pyrethroid resistant and susceptible Anopheles gambiae mosquitoes to insecticide treated bed net
Source: PLoS One. 2022 Apr 7;17(4):e0266420. doi: 10.1371/journal.pone.0266420 (PMC8989192; doi:10.1371/journal.pone.0266420)
Supplement: S3 Table — (DOCX) [file pone.0266420.s003.docx]

**S3 Table. Summary results on Bioefficacy of deltamethrin treated against pyrethroid resistant and susceptible *Anopheles gambiae* mosquitoes.**

| Progeny | Species | age | ITN type | No. Exposed | KD 00 min | 3 mins | KD 30Min | KD60MIN | KD AFTER 24HR | Total alive |
| --- | --- | --- | --- | --- | --- | --- | --- | --- | --- | --- |
| S-F13.1 | An.gambiae | 3-4 days | LLIN (Dawa plus 2) | 120 | 0 | 0 | 35 | 77 | 111 | 9 |
| S-F13.2 | An.gambiae | 3-4 days | LLIN (Dawa plus 2) | 120 | 0 | 0 | 21 | 69 | 110 | 10 |
| S-F13.3 | An.gambiae | 3-4 days | LLIN (Dawa plus 2) | 120 | 0 | 2 | 47 | 75 | 111 | 9 |
| S-F13.1 | An.gambiae | 3-4 days | PermNet 3.0 | 120 | 0 | 42 | 119 | 120 | 120 | 0 |
| S-F13.2 | An.gambiae | 3-4 days | PermNet 3.0 | 120 | 0 | 39 | 119 | 120 | 120 | 0 |
| S-F13.3 | An.gambiae | 3-4 days | PermNet 3.0 | 120 | 0 | 35 | 120 | 120 | 120 | 0 |
| R-F4.1 | An.gambiae | 3-4 days | LLIN (Dawa plus 2) | 120 | 0 | 0 | 1 | 10 | 16 | 104 |
| R-F4.2 | An.gambiae | 3-4 days | LLIN (Dawa plus 2) | 120 | 0 | 0 | 1 | 10 | 21 | 99 |
| R-F4.3 | An.gambiae | 3-4 days | LLIN (Dawa plus 2) | 120 | 0 | 0 | 1 | 3 | 8 | 112 |
| R-F4.1 | An.gambiae | 3-4 days | PermNet 3.0 | 120 | **0** | 5 | 103 | 116 | 118 | 2 |
| R-F4.2 | An.gambiae | 3-4 days | PermNet 3.0 | 120 | 0 | 4 | 93 | 112 | 119 | 1 |
| R-F4.3 | An.gambiae | 3-4 days | PermNet 3.0 | 120 | 0 | 8 | 96 | 115 | 120 | 0 |
| wild population | An.gambiae s.s | | Dawa plus 2 | 120 | 0 | 10 | 30 | 43 | 71 | 49 |
| wild population | An.gambiae s.s | | PermNet 3.0 | 120 | 0 | 46 | 113 | 118 | 120 | 0 |
| lab strain | An.gambiae s.s | | Dawa plus 2 | 120 | 0 | 24 | 76 | 115 | 120 | 0 |
| lab strain | An.gambiae s.s | | PermNet 3.0 | 120 | 0 | 61 | 118 | 120 | 120 | 0 |
